# An Unusual Case of a Giant Schwannoma of the Sciatic Nerve: A Case Report With a Review of Literature

**DOI:** 10.7759/cureus.51155

**Published:** 2023-12-27

**Authors:** Ousama Jelti, Oussama El Alaoui, Adnane Lachkar, Najib Abdeljaouad, Hicham Yacoubi

**Affiliations:** 1 Orthopedics, Mohammed VI University Hospital, Oujda, MAR; 2 Orthopedics and Traumatology, Centre Hospitalier Universitaire (CHU) Mohammed VI Oujda, Oujda, MAR; 3 Department of Traumatology and Orthopedic, Mohammed VI University Hospital, Oujda, MAR; 4 Faculty of Medicine and Pharmacy, Mohammed First University, Oujda, MAR

**Keywords:** surgery, case report, neurilemmoma, schwannoma, sciatic nerve

## Abstract

Sciatic nerve schwannomas are rare tumors, mainly characterized by sciatic neuralgia rather than sensory-motor deficits. The poorly suggestive clinical presentation of this localization leads to a delayed diagnosis. Here, we describe the case of a 47-year-old female patient with a nine-month history of schwannoma localized in the sciatic nerve, just above the left popliteal fossa. Although magnetic resonance imaging (MRI) is the imaging modality of choice, the final diagnosis rests on the histological examination of the tumor. The schwannoma must be surgically removed without severing the nerve trunk.

## Introduction

Benign tumors affecting peripheral nerves represent a small subset of soft tissue neoplasms. Schwannomas, originating from Schwann cells, emerge as the predominant category of peripheral nerve tumors [1]. Initially described in the early 19th century, these tumors are more commonly observed between the ages of 30 and 60, with a balanced gender distribution [2]. While the isolated form is prevalent, there are instances of multiple occurrences, known as schwannomatosis, affecting the same limb segment [3]. These tumors are encapsulated, well-demarcated, and located outside the nerve pathways, displacing nerve fascicles. Despite potentially substantial lesion sizes, the presence of neurological symptoms is limited, explaining the rarity of malignant transformation [4]. Schwannomas typically favor large nerve trunks, especially in the context of the upper limbs, with the posterior tibial nerve being the most frequently affected in the lower limbs [5]. Localization in the sciatic nerve is uncommon, affecting approximately 1% of patients [6]. The misleading clinical presentation of this localization can lead to diagnostic delays. Although magnetic resonance imaging (MRI) is the preferred imaging modality, the conclusive diagnosis relies on a histological examination of the tumor.

## Case presentation

**Figure 1:**
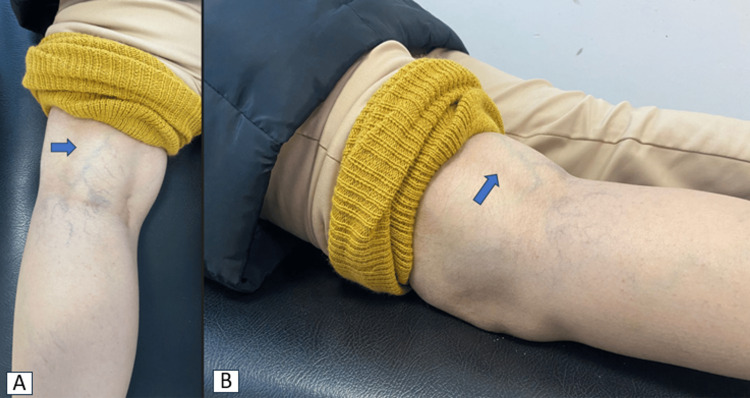
Clinical picture of the tumor.

We describe the case of a 47-year-old woman with no pathological history who had been suffering for nine months from pain along the left lower limb, associated with a painful mass located immediately above the popliteal fossa. Physical examination revealed a mobile mass above the left popliteal fossa, while knee mobility was normal. No neurological or vascular deficits were observed (Figure 1). Three initial potential differential diagnoses were entertained before imaging studies. The mobile mass prompted consideration of a lipoma, a benign fatty tumor. The possibility of a Baker's cyst, characterized by synovial fluid accumulation, was also raised due to the painful mass in the popliteal region. Moreover, the persistent pain and mobility of the mass led to consideration of a soft tissue tumor, such as a sarcoma.

**Figure 2:**
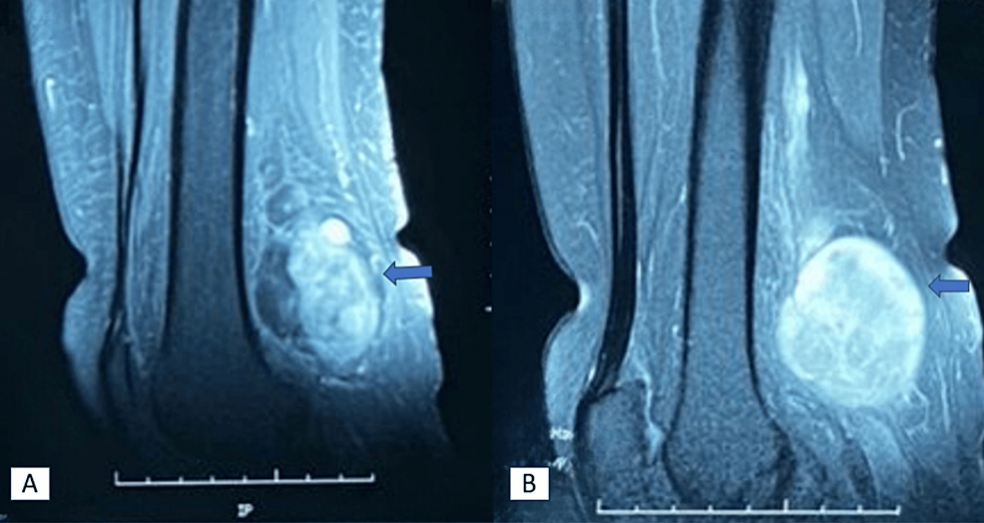
MRI scan showing a fusiform mass measuring 32x42x72 mm in the path of the sciatic nerve.

MRI revealed a smoothly contoured, roughly fusiform formation located on the course of the common fibular nerve with heterogeneous T2 signal, with central T2-hyposignal areas surrounded by a peripheral corona of T2 and T2 fat-saturation (FS) free hypersignal, T1-hyposignal, and free diffusion hypersignal without apparent diffusion coefficient (ADC) drop with central and nodular enhancement measuring 32 x 42 x 72 mm (Figure 2).

**Figure 3:**
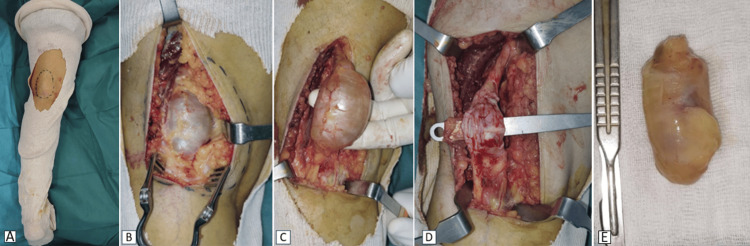
Intraoperative images of tumor resection.

The patient was positioned prone under spinal anesthesia. A longitudinal incision was made in the left popliteal fossa. A clearly circumscribed tumor was identified, emanating from the proximal sciatic nerve at its bifurcation. The tumor was completely excised while preserving the functional fascicles. The tumor was whitish in color, encapsulated, in direct contact with the sciatic nerve, and located eccentrically, facilitating its excision (Figure 3).

**Figure 4:**
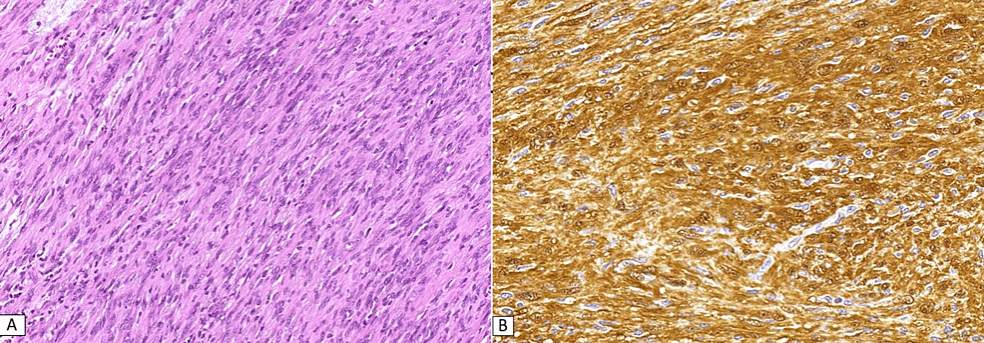
A microphotograph of the lesion shows diffuse proliferation arranged in multiple intersecting fascicles; tumor cells are spindle-shaped, displaying bland and elongated nuclei with fine chromatin and an eosinophilic cytoplasm (A). Immunohistochemical examination shows diffuse and strong staining for S100 (B).

Histological analysis of the tumor showed encapsulated tumor proliferation arranged in two areas: Antoni A has dense cellularity made up of regular spindle cells without atypia or mitosis, arranged in a palisade pattern. Antoni B was loosely cellular. Vessels were abundant, dilated, and hyalinized, with a few lymphocytic elements. An immunohistochemical study showed strong, diffuse PS100 staining of tumor cells, with no CD34 or AML staining (Figure 4).

No signs of sensitivomotor deficits were observed during post-operative follow-up, and the pain initially reported by the patient disappeared completely. With a 24-month follow-up, no tumor recurrence was noted.

## Discussion

Schwannomas represent the predominant category of peripheral nerve tumors [1], having been initially described in the early 19th century [2]. Although these tumors can occur at any age, they are most frequent between the ages of 30 and 60, with a balanced sex ratio [2]. The isolated form remains the most common, but there are multiple occurrences, known as schwannomatosis, that affect the same limb segment [3]. These tumors are encapsulated and clearly delimited and lie outside the nerve pathway, pushing back the nerve fascicles. This characteristic explains why, despite the sometimes large size of the lesions, there are few neurological symptoms. Malignant transformation of these tumors remains rare [4]. Schwannomas generally have a preference for large nerve trunks, especially in the context of the upper limbs. In the lower limb, the nerve most frequently affected is the posterior tibial nerve [5]. Localization in the sciatic nerve is uncommon, occurring in around 1% of patients [6-9]. It is possible for the tumor to develop along the entire course of the nerve [10]. Irrespective of the tumor site, clinical examination is aimed at detecting the presence of pain, swelling, and/or a possible irritative syndrome [2,11]. Schwannomas are generally single, medium-sized, slow-growing tumors which may be palpable when large or located on the surface. Symptomatology develops progressively, manifesting as paresthesia-like pain, often the first perceptible sign [12], rarely accompanied by a motor or sensory deficit.

On ultrasound, a schwannoma appears as a hypoechoic mass, eccentric to the nerve, which retains its fibrillar structure. A neurofibroma is an alternative diagnosis. Unlike schwannoma, it presents as a solid mass well centered in relation to the supporting nerve, with complete disappearance of the fibrillar structure [13,14]. MRI remains the gold standard for diagnosing schwannomas, showing them as masses eccentric to the nerve or root tract, with an isointense signal in T1 and a hyperintense signal in T2 [11]. MRI can be used to distinguish schwannoma from neurofibroma, a crucial distinction given that the invasive aspect of neurofibroma makes dissection of the tumor difficult, leading to a significant risk of sequelae and recurrence. A confirmatory diagnosis is based on histology [15].

As a general rule, the preferred treatment for schwannomas is surgery. These tumors are theoretically extirpable, as they push back the fascicular groups without infiltrating them. The method of choice for treating these extirpable tumors is microsurgical enucleation, using electrical stimulation to localize the motor fascicles and including meticulous dissection of the surrounding nerve bundles [16,17]. This aims to maintain nerve fiber continuity, leading to favorable results with a very low recurrence rate [11,18]. If these dissection criteria are respected, postoperative neurological deficits are rare, with a rate of less than 15%, generally transient, and explained by contusion of the nerve fibers that remain intact [19]. In a study of 14 cases, Akambi et al. [15] had to resect nerve fascicles with the tumor in eight cases, and three of these patients retained a sensory deficit after an average delay of eight months. The persistence of clinical symptoms should arouse suspicion of another undetected localization, given the small size of the tumor, and a systematic search should be undertaken at the time of surgery [11]. When deficit signs are present before surgery, their regression or stabilization will depend on the size and age of the lesion [4,20].

## Conclusions

Extirpable peripheral nerve tumors, such as schwannomas, have a generally favorable prognosis when appropriately treated surgically while preserving the nerve fascicles, irrespective of their location. The recurrence rate is minimal and may be ascribed to incomplete surgical resection during the initial management. The presence of persistent sciatica despite a negative routine evaluation should raise suspicion of peripheral nerve neoplasms. While MRI is the preferred radiological examination, the conclusive diagnosis relies on the histological examination of the tumor.
